# The psychometric properties of the German version of the WHOQOL-OLD in the German population aged 60 and older

**DOI:** 10.1186/s12955-014-0105-4

**Published:** 2014-09-05

**Authors:** Ines Conrad, Herbert Matschinger, Steffi Riedel-Heller, Carolin von Gottberg, Reinhold Kilian

**Affiliations:** Institute for Social Medicine, Occupational Health and Public Health, University of Leipzig, Medical Faculty, Philipp-Rosenthal-Str. 55, 04103 Leipzig, Germany; Institute of Health Economics and Health Service Research, University of Hamburg, Hamburg Center for Health Economics, Martinistr. 52, 20246 Hamburg, Germany; Department of Psychiatry and Psychotherapy II, Ulm University, Ludwig-Heilmeyer-Str. 2, 89312 Günzburg, Germany

**Keywords:** Quality of life, Old age, WHOQOL-OLD, Representative survey, Psychometric properties

## Abstract

**Background:**

The WHOQOL-OLD is an instrument for the assessment of subjective quality of life in elderly people. It is based on the WHO definition of quality of life and is available in more than 20 languages. However, in most countries, the psychometric properties of the WHOQOL-OLD have been assessed only on the basis of small local samples and not in representative studies. In this study, the psychometric properties of the WHOQOL-OLD are evaluated based on a representative sample of Germany’s elderly population.

**Methods:**

Face-to-face interviews with 1133 respondents from the German population aged 60 years and older were conducted. Quality of life was assessed by means of the WHOQOL-BREF, the WHOQOL-OLD and the SF12. Moreover, the GDS, the DemTect and the IADL were applied for the assessment of depressive symptoms, cognitive capacities and capacity for carrying out daily activities. Psychometric properties of the WHOQOL-OLD were evaluated by means of classical and probabilistic test theory, confirmatory factor analysis and multivariate regression model.

**Results:**

Cronbach’s alpha was found to be above 0.85 for four and above .75 for two of the six facets of the WHOQOL-OLD. IRT analyses indicated that all items of the WHOQOL-OLD contribute considerably to the measurement of the associated facets. While the six-facet structure of the WHOQOL-OLD was well supported by the results of the confirmatory factor analysis, a common latent factor for the WHOQOL-OLD total scale could not be identified. Correlations with other quality of life measures and multivariate regression models with GDS, IADL and the DemTect indicate a good criterion validity of all six WHOQOL-OLD facets.

**Conclusions:**

Study results confirm that the good psychometric properties of the WHOQOL-OLD that have been found in international studies could be replicated in a representative study of the German population. These results suggest that the WHOQOL-OLD is an instrument that is well suited to identify the needs and the wishes of an aging population.

## Introduction

Given the predictions of an aging population, assessment of quality of life (QoL) of older adults is increasingly important. People in Europe are older than people in any other world region, and older adults are expected to increase to 25% of the population in several European countries by 2020 [1]. In the United States, 12% of the population or 36.3 million people are over the age of 65 years. It is projected that by 2050, 21% of the American population will be over 65 [2].

The changing demographics have significant implications for policy makers, as well as professionals providing health and social services [3]. As a result of higher life expectancy and a trend to earlier retirement, many people in industrialized societies spend an increasing proportion of their lifetime in the “third age” [4]; that is the stage of life between retirement and the age when 50% of the age group have died [5]. Variable life courses as well as social, economic and political conditions [4,5] result in a great variety of health states and living conditions.

Although QoL measurement is becoming increasingly important, issues exist regarding measurement in older adults. There is a lack of age-specific measurements [6], and the appropriateness of QoL instruments designed for younger adults has been questioned [6-8].

There have been a number of conceptualizations of QoL for older adults. Using Erikson’s theory of life cycles [9,10], we define QoL in later life as the capacity to satisfy higher order needs of Maslow’s hierarchy, in particular control, autonomy, pleasure and self realization. While both approaches appropriately separate QoL from the environmental and intra-personal factors that influence it, they are limited because they ignore the subjective experience [11] and use a deductive approach to identifying the dimensions of QoL. Moreover, concepts such as control, autonomy, pleasure and self-realization may be more relevant to Western cultures. Since QoL is regarded as a universal concept reflecting the subjective experience of people [12], the individual experience, as well as cultural differences, must be taken into consideration. Consequently, the WHOQOL-OLD, an add on-module of the younger adults version of the WHOQOL for use with older adults, was developed in a cross-cultural study.

In 1991, the World Health Organization Quality of Life Project (WHOQOL) was the first attempt to take account of cultural differences during the instrument development [13-15]. This was based on the following definition of quality of life: “*Quality of life is defined as individuals’ perceptions of their position in life in the context of the culture and value systems in which they live and in relation to their goals, expectations, standards and concerns*”. The center of this definition is the subjective perception und evaluation of the living conditions by the individual. Furthermore, as a fundamental characteristic of this approach, the term quality of life is embedded into an intercultural context [13,14,16,17]. The intercultural comparability of the WHOQOL-OLD instrument, it was claimed, was ensured by the participation of research centers from diverse cultural areas in the development of the pilot instrument. This included the definition and operationalization of individual facets (sub-categories) and facets (main categories) of quality of life, the formulation and choice of questionnaire items, the development of response scales for single item groups and the field testing of the instrument by conducting pilot studies [12,18]. The result of this WHOQOL-project was the development of two generic instruments for the assessment of quality of life: the WHOQOL-100 and its short form WHOQOL-BREF [13-15,19]. Today, these two instruments are available in approximately 30 languages [19]. However, it became more and more obvious that the generic version of the WHOQOL-questionnaires was insufficient for the specific requirements of the assessment of quality of life in old age.

**Figure 1:**
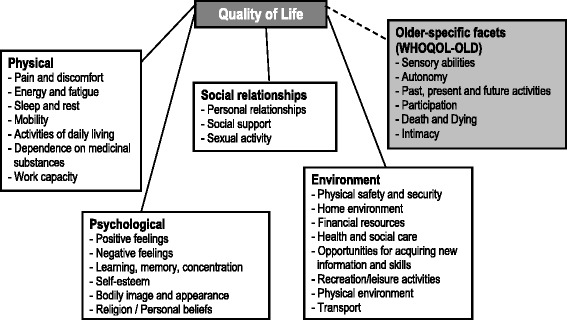
**Dimensions of quality of life - WHOQOL-BREF & WHOQOL-OLD (older-specific facets).**

Therefore, a worldwide project called WHOQOL-OLD for the development of an instrument for the intercultural assessment of quality of life in old age, based on the WHOQOL-100 was initiated. Within the scope of this project, under the patronage of the WHO, research centers from 22 countries developed an instrument for the assessment of quality of life in old age. In order to determine the dimensional structure of the quality of life concept for older people, as well as to develop facet definitions, focus groups with experts and lay persons were conducted at project baseline. The results showed that older people relate the term quality of life to social, health-related and environmental aspects [20]. Based on these results, items were generated whose psychometric characteristics were evaluated by a pilot study. The results of this study led to a reduction of items. The psychometric verification of the questionnaire was carried out within a survey among the respective age target-group population. The result of this study was the final version of the WHOQOL-OLD questionnaire for the assessment of quality of life in older people, consisting of six new facets (Figure 1), which can be applied in combination with the WHOQOL-100 or the WHOQOL-BREF, respectively. However, the calculations of the psychometric characteristics of the final version of this instrument for the assessment of quality of life in older people (WHOQOL-OLD) were based on the same data set that was used for the development of the final version. Although the WHOQOL-OLD exists in more than 20 languages, validation of the instrument in general populations is rare. Only recently a Chinese version has been evaluated in the general population of Guangzhou (formerly Canton) [21].

In this article, the psychometric properties of the German version of the WHOQOL-OLD are assessed on the basis of a representative survey of the German population aged 60 years and older.

## Methods

### Data

In 2012, a representative, face-to-face survey of respondents 60 years and older was conducted in Germany. The sample was drawn using a random sampling procedure with three stages: (1) sample points (regional area), (2) households, and (3) individuals within the target households. Target households within 129 sample points were determined according to the random route procedure. 105 sample points comprise the area of the old and 24 the area of the new “Länder” of Germany. Target persons were selected using random digits. For the 129 sample points, a gross N of 5418 was chosen in order to finally realize a total sample of about 1000 respondents. In a second step for the age group 8o+, an additional sample was drawn in order to increase this part of the sample to about 300. Adding this sample of 102 respondents to the first one resulted in a total of 1133 (309 respondents aged 80 and older).

Ethical approval was obtained (University of Leipzig and Ulm University).

### Instruments

To control for the inability to conduct the interview, the interview began with the DemTect in order to identify respondents with severe cognitive impairment. The DemTect is a cognitive screening test (including 5 tasks: a word list, a number transcoding task, a word fluency task, digit span reverse, delayed recall of the word list) to support the diagnosis of Mild Cognitive Impairment (MCI) and early dementia. Its transformed total score is independent of age and education [22].

- Total score 13-18: Cognitive powers appropriate for subject’s age
- Total score 9-12: Mild cognitive impairment
- Total score 0-8: Suspected dementia.

To assess subjective QoL, the German version of the WHOQOL-BREF (Figure 1) consisting of the six domains: “physical” (7 items), “psychological” (6 items), “social relationships” (3 items), “environment” (8 items) and “overall QoL” (2 items) was used [16,19]. Values of domains will be transformed into a range between 0 and 100. Internal consistency, as measured with Cronbach’s alphas, of all subscales ranged between 0.57 and 0.88. For assessing older-specific facets of quality of life, the 24-item add-on module, WHOQOL-OLD, consisting of 6 facets (*sensory abilities*, *autonomy*, *past, present and future activities*, *social participation*, *death and dying and intimacy*) was used (Figure 1, Table 1) [23]. Values of facets were transformed into a range between 0 and 100, as well. Internal consistency of the subscales ranged between 0.75 for *autonomy* and 0.92 for *intimacy*.

**Table 1:** **The structure of the WHOQOL-OLD**

**WHOQOL-OLD facet**	**Item no.**	**Item text**
	Old 01	Impairments to senses affect daily life
	Old 02	Rate sensory functioning
I: Sensory abilities	Old 10	Loss of sensory abilities affect participation in activities
	Old 20	Problems with sensory functioning affect ability to interact
	Old 03	Freedom to make own decisions
	Old 04	Feel in control of your future
II: Autonomy	Old 05	Able to do things you’d like to
	Old 11	People around you are respectful of your freedom
	Old 12	Happy with things to look forward to
	Old 13	Satisfied with opportunities to continue achieving
III: Past, present and future activities	Old 15	Received the recognition you deserve in life
	Old 19	Satisfied with what you’ve achieved in life
	Old 14	Satisfied with the way you use your time
	Old 16	Satisfied with level of activity
IV: Social participation	Old 17	Have enough to do each day
	Old 18	Satisfied with opportunity to participate in community
	Old 06	Concerned about the way you will die
V: Death and dying	Old 07	Afraid of not being able to control death
	Old 08	Scared of dying
	Old 09	Fear pain before death
	Old 21	Feel a sense of companionship in life
	Old 22	Experience love in your life
VI: Intimacy	Old 23	Opportunities to love
	Old 24	Opportunities to be loved

Comorbidity was defined as the number of chronic diseases using the comorbidity list from the Federal Health Survey [24].

The respondents’ functioning level concerning instrumental activities of daily living (IADL) was assessed with the Instrumental Activities of Daily Living Scale [25,26].

### Statistical analysis

#### Assessment of reliability

According to recent developments in psychometric assessment in quality of life research [27-30], psychometric properties of WHOQOL-OLD were assessed by means of classical and probabilistic test theory [8].

Following the principles of the classical test theory, the reliability of the WHOQOL-OLD facets was determined on the basis of the internal consistency. Cronbach’s alpha was estimated for the 6 facets of the WHOQOL-OLD. The inter item correlation as well as the item scale correlation were estimated for all items in relation to the 6 facets.

To examine the reliability by means of probabilistic test theory, a **P**artial **C**redit **M**odel (**PCM**) was employed [31-33]. The **PCM** comes from the family of IRT (Item Response Theory) models, and is an extension of the Rasch model [34,35] for polytomous items with ordered response categories:

$$ P\left({y}_{ip}=j\Big|{\theta}_p,{\delta}_{il}\right)=\frac{ \exp {\displaystyle \sum_{l=0}^j\left({\theta}_p-{\delta}_{il}\right)}}{{\displaystyle \sum_{k=0}^{m_i} \exp {\displaystyle \sum_{l=0}^k\left({\theta}_p-{\delta}_{il}\right)}}}\kern4em {\displaystyle \sum_{l=0}^k\Big({\theta}_p-{\delta}_{il}}\Big)=0 $$

The **PCM** models the probability of response category *j* for item *i* and person as a function of the latent “ability” *θ*_*p*_ and the threshold parameter *δ*_*il*_ [36]. Both the thresholds and the latent ability are mapped on the same scale. The threshold parameters mark the point on the latent dimension *θ* where the **C**ategory **C**haracteristic **C**urves intersect (e.g. the point where the probability of endorsing 2 particular adjacent categories is equal). Whether the thresholds are located on the dimension in ascending order is of major concern and not a necessary characteristic of this (ordinal) model. The **PCM** is suited to model sums of binary responses which are not supposed to be stochastically independent [37].

To evaluate model, two fit-indices were estimated. First, “INFIT” and “OUTFIT” which are measures for the “randomness” or “determination” of an item concerning a particular measurement model were estimated. “Values larger than 1.0 indicate unmodeled noise. Values are on a ratio scale, so that 1.2 indicates 20% excess noise. Values less than 1.0 indicate a lack of stochasticity” [33,38-41]. Since the INFIT is an information-weighted form of the OUTFIT which “…reduces the influence of less informative, low variance, off-target responses” [38], we expressly will focus on this parameter. This leads to the pragmatic categorization [42]:

- > 2.0 Distorts or degrades the measurement system
- 1.5 - 2.0 Unproductive for construction of measurement, but not degrading
- 0.5 - 1.5 Productive for measurement
- < 0.5 Less productive for measurement, but not degrading. May produce misleadingly good reliabilities and separations.

Secondly, the so-called Q-index (also called Person-Separation-Index **PSI**) [43,44] was estimated. “The Q-index lies between zero (indicating perfect discrimination, i.e., a Guttman-pattern) and one (indicating perfect “anti-discrimination”). A value of 0.5 indicates no relationship between the individual parameter and the reaction to the item. The *Zq* value is a transformation of the Q-index that is approximately normally distributed if the Rasch model holds for the respective item. High positive values indicate that the item discrimination is lower than assumed by the Rasch model (under-fit), negative values indicate higher discrimination than assumed (over-fit)” [45]. ZQ values within the range of -1.96 and 1.96 indicate that the Q-index of an item is in the expected range with a probability of 95%.

The thresholds for the answering categories and the distributions of the latent scale dimensions are presented in the person-item maps (PIM). The histograms in the upper part of the PIM represent the distribution on the latent scale of each facet. The lines in the lower part of the PIM represent the ranges of the latent scales with the means symbolized, as dark dots and the thresholds of the k-1 answering categories symbolized as circles with the number of the category. As an indicator of a high reliability, all thresholds should have the same ascending order. The discriminatory power of the items is represented by the range between the thresholds. Small ranges represent a high discriminatory power and vice versa. Since the PCM supposes an ordinal scaling model, it does not require equal ranges between thresholds.

### Assessment of validity

The construct validity of the WHOQOL-OLD was assessed by means of a first order and second order confirmatory factor analysis. The first model represents the 6 factor structure in the sense of a congeneric measurement model [46]. The second model contains an additional factor of 2^nd^ order that was included to investigate whether the construct “Quality of Life” might be represented by one single dimension.

Convergent validity of the WHOQOL-OLD is determined by examining the correlations between the WHOQOL-OLD facets and a set of criterion variables. Criterion variables include generic quality of life measured by the WHOQOL-BREF and the SF12 Physical Health Index (SF12 PHI) and the SF12 Mental Health Index (SF12 MHI).

Discriminant validity was assessed by multivariate regression models for each of the WHOQOL-OLD facets with the socio-demographic characteristics, the living situation, the GDS, the IADL, the number of chronic diseases and the cognitive status measured by the DemTect as independent variables.

### Software

The CFA was estimated by **Mplus 7.11** [47]. Analyses for the PCM were conducted by the package **eRm** [48] or **ltm** [49] for **R**. The Q-Index was computed using **WINMIRA** [45]. The indices regarding “classical test theory” were estimated by the command “**alpha**” using **STATA 13** [50].

## Results

A total of 1133 people aged 60 to 96 years old participated in the study (Table 2). The mean age was 72.3 years (SD 8.7 years). The gender ratio of the sample was about equal. About 50% of the sample was married and lived together with a spouse, while the other half were separated, divorced, widowed or never married. About 43% of the study population lived alone, while 57% lived together with partners, children or other people. About 42% of the study population had finished ten years or more of formal education, while 58% had finished less than 10 years of school.

**Table 2:** **Sample characteristics**

**N**		**1133**
**Age mean (SD)**		72.5 (8.7)
**Gender n (%)**	Female	616 (54.4)
**Family status n (%)**	Married	559 (49.3)
	Separated, divorced, widowed, never married	574 (50.7)
**Living arrangement (%)**	Alone	483 (42.6)
	With others	654 (57.4)
**Education (%)**	High	479 (42.3)
	Basic	654 (57.7)
**DemTect categories n (%)**	0 severe impairments	104 (9.5)
	1 mild impairments	269 (24.3)
	2 no impairments	730 (66.3)
**Number of chronic diseases mean (SD)**		5.3 (3.8)
**IADL mean (SD)**		6.7 (1.7)
**GDS mean (SD)**		3.4 (3.8)

Of the study population 66% had no cognitive impairments, 24% had mild impairments and 9.5% were identified as having severe cognitive impairments according to the DemTect.

The mean Instrumental Activity of Daily Living (IADL) score is 6.7, indicating that the study participants, on average, are able to live largely independent. The mean Geriatric Depression Scale (GDS) value of 3.5 indicates a low level of depressive symptoms.

### Reliability

Table 3 shows the reliability parameters for the WHOQOL-OLD facets according to classical and probabilistic test theory. Cronbach’s alpha (α) indicates a high reliability for the WHOQOL-OLD facets *sensory abilities* (α = 0.8842), *social participation* (α = 0.8502), *death and dying* (α = 0.8567), and *intimacy* (α = 0.9162), and a sufficient reliability for the facets *autonomy* (α = 0.7537) and *past and present activities* (α = 0.7619). The corrected item test correlations are above the critical value of 0.3 for all items. The mean inter-item correlations are between 0.4015 for the facet *autonomy* and 0.7324 for the facet *intimacy* indicating a high homogeneity of the WHOQOL-OLD items.

**Table 3:** **Reliability parameters of the WHOQOL-OLD facets**

	**Classical test theory**				**IRT**	
**Item no./WHOQOL-OLD facet**	**Item-test correlation**	**Corrected item-test correlation**	**Inter-item correlation**	**Cronbach’s alpha if item deleted/alpha**	**Q-Index**	**INFIT**
Old 01	0.8882	0.7849	0.6350	0.8369	−0.1828	0.687
Old 02	0.9137	0.8337	0.6035	0.8167	−0.5315	0.509
Old 10	0.8206	0.6755	0.7097	0.8793	0.6041	1.031
Old 20	0.8255	0.7114	0.6842	0.8669	0.1172	0.780
**Sensory abilities**			*0.6581*	*0.8842*	**Andrich reliability**	
					0.798	
Old 03	0.7790	0.6062	0.4057	0.6696	−0.5757	0.662
Old 04	0.7595	0.5148	0.4694	0.7218	0.5710	0.847
Old 05	0.7472	0.5558	0.4371	0.6949	−0.0180	0.747
Old 11	0.7594	0.5409	0.4519	0.7017	0.1018	0.797
**Autonomy**			*0.4015*	*0.7537*	**Andrich reliability**	
					0.703	
Old 12	0.8197	0.6062	0.4228	0.6851	−0.1279	0.694
Old 13	0.7849	0.5811	0.4377	0.6945	−0.0293	0.737
Old 15	0.7141	0.5254	0.4750	0.7263	0.2049	0.804
Old 19	0.7402	0.5551	0.4608	0.7112	−0.0690	0.771
**Past, present and future activities**			*0.4491*	*0.7619*	**Andrich reliability**	
					0.751	
Old 14	0.7514	0.5667	0.6817	0.8596	1.3272	1.071
Old 16	0.8309	0.7167	0.5781	0.8042	−0.4557	0.694
Old 17	0.8897	0.7891	0.5318	0.7656	−0.9818	0.540
Old 18	0.8600	0.7146	0.5804	0.8019	0.0255	0.718
**Social participation**			*0.5930*	*0.8502*	**Andrich reliability**	
					0.801	
Old 06	0.8443	0.7211	0.5859	0.8095	−0.5266	0.737
Old 07	0.8806	0.7666	0.5559	0.7884	−1.2724	0.610
Old 08	0.8163	0.6747	0.6163	0.8280	0.4899	0.849
Old 09	0.8044	0.6432	0.6403	0.8415	1.1852	0.946
**Death and dying**			*0.5996*	*0.8567*	**Andrich reliability**	
					0.829	
Old 21	0.8675	0.7667	0.7625	0.9051	0.5563	0.901
Old 22	0.9230	0.8517	0.7022	0.8767	−0.5745	0.578
Old 23	0.8958	0.8153	0.7278	0.8891	−0.1093	0.706
Old 24	0.8897	0.8018	0.7369	0.8934	0.1258	0.765
**Intimacy**			*0.7324*	*0.9162*	**Andrich reliability**	
					0.888	

Reliability coefficients from the IRT partial credit model reveal a good reliability (Andrich reliability) for the facets *social participation* (0.801), *death and dying* (0.829) and *intimacy* (0.888) and a sufficient reliability for the facets *sensory abilities* (0.798), *autonomy* (0.703) and *past, present and future activities* (0.751).

The INFIT parameters between 0.5 and 1.5 indicate that all items are productive for the measurement of the associated facets. The z values for the transformed Q-index indicate no significant deviance of the response patterns from those expected by the partial credit model.

**Figure 2:**
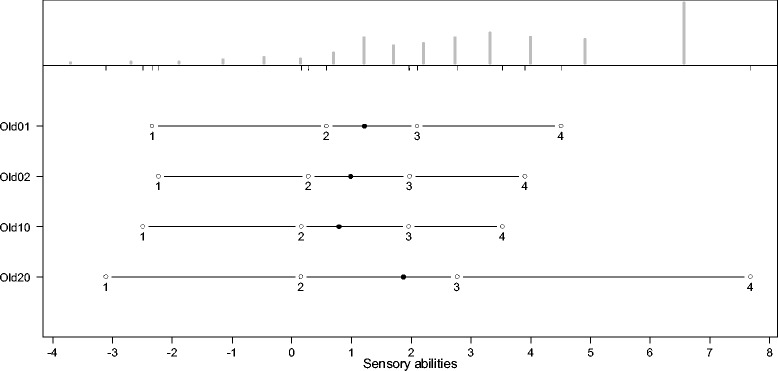
**Person-item map (PIM) of the WHOQOL-OLD facet “sensory abilities”.**

**Figure 3:**
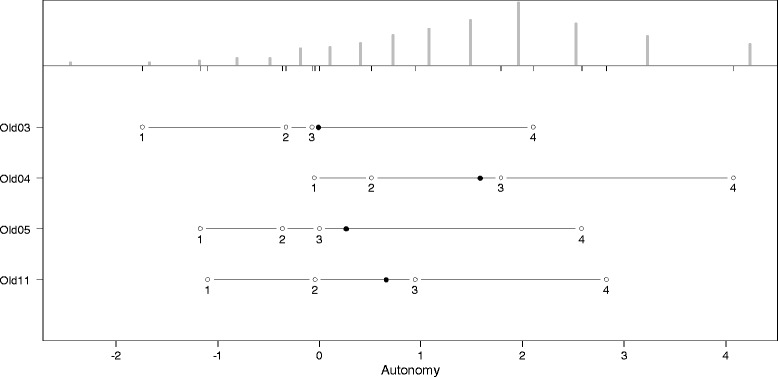
**Person-item map (PIM) of the WHOQOL-OLD facet “autonomy”.**

**Figure 4:**
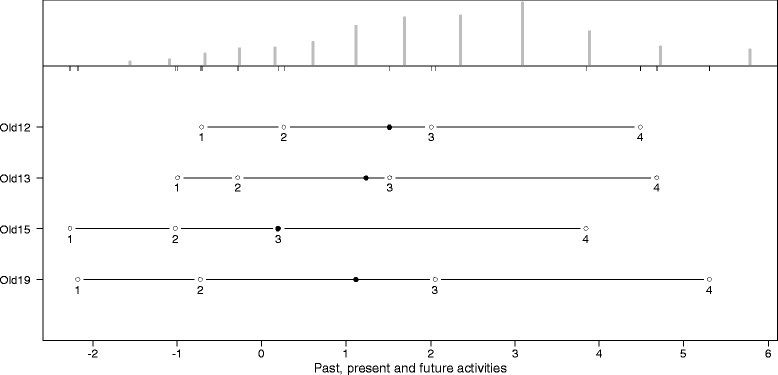
**Person-item map (PIM) of the WHOQOL-OLD facet “past, present and future activities”.**

**Figure 5:**
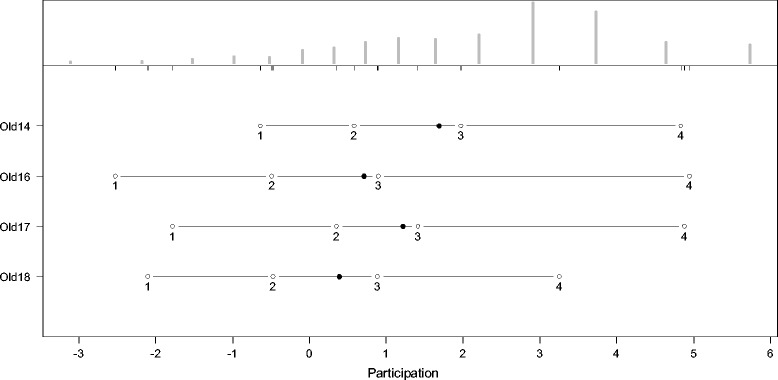
**Person-item map (PIM) of the WHOQOL-OLD facet “social participation”.**

**Figure 6:**
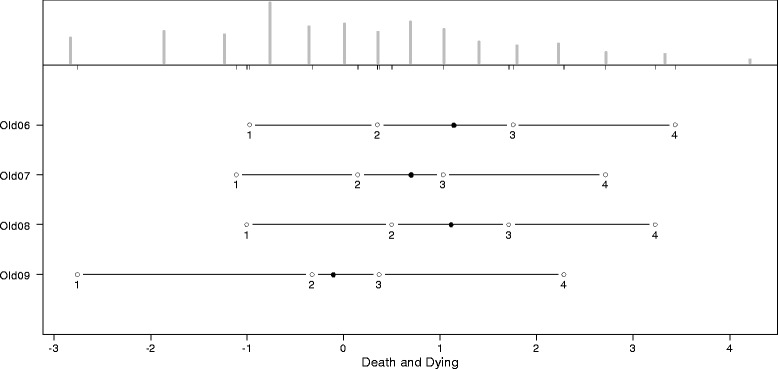
**Person-item map (PIM) of the WHOQOL-OLD facet “death and dying”.**

**Figure 7:**
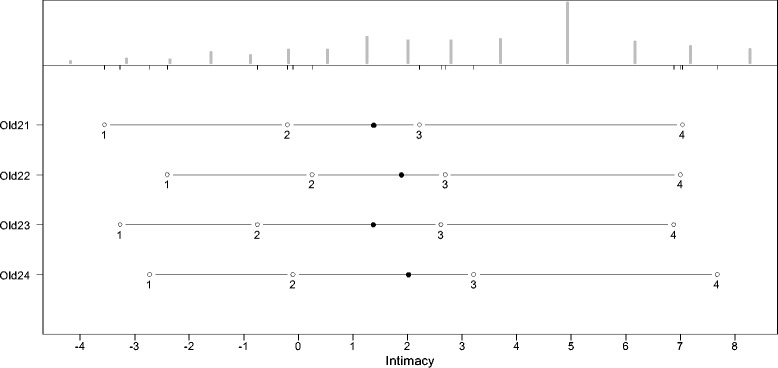
**Person-item map (PIM) of the WHOQOL-OLD facet “intimacy”.**

As indicated by Figures 2, 3, 4, 5, 6 and 7, all facets show ordered answering thresholds for the associated items. The varying threshold ranges within and between the items of each facet indicate considerable differences in the discriminatory power not only of the items but also of the answering categories within the same items.

Frequency distributions for the latent scales indicate negatively skewed distributions for all facets, however the modal value of the facet *death and dying* is much lower than those of the other facets. Particularly the facet *sensory abilities* but also *death and dying* have bimodal distributions.

### Validity

#### Construct validity

**Figure 8:**
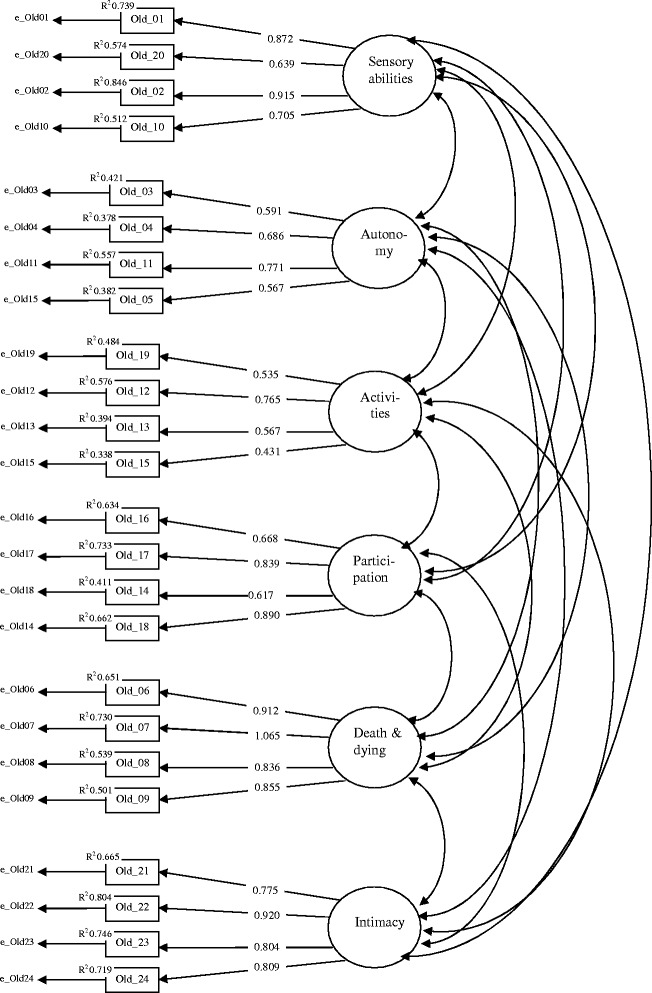
**Confirmatory factor model for the six WHOQOL-OLD facets (standardized loadings).**

Results of the first order confirmatory factor analysis (Figure 8) reveal that all WHOQOL-OLD facets are represented by sufficient significant standardized loadings above 0.5 on the associated items. The only exception is the small loading of the facet *past present and future activities* on the item 19 (0.339). R^2^ values indicate sufficient communalities of above 0.3 for all items with the exception of item 19 with 0.431.

As shown in Table 4, correlations between the factors representing the 6 WHOQOL-OLD facets range between r = 0.180 between *sensory abilities* and *death and dying* and r = 0.907 between *social participation* and *past, present and future activities*. In particular, the high correlations between the factors representing the facets *social participation*, *autonomy* and *past, present and future activities* suggest that a latent common factor representing a WHOQOL-OLD total score may exist.

**Table 4:** **Inter-correlations of the factors representing the WHOQOL-OLD facets**

	**Sensory abilities**	**Autonomy**	**Past, present and future activities**	**Social participation**	**Death and dying**
**Autonomy**	0.491***				
**Past, present and future activities**	0.466***	0.900***			
**Social participation**	0.487***	0.808***	0.907***		
**Death and dying**	0.180***	0.202***	0.323***	0.229***	
**Intimacy**	0.323***	0.576***	0.705***	0.557***	0.252***

*p ≤ 0.05, **p ≤ 0.01, ***p ≤ 0.001.

**Figure 9:**
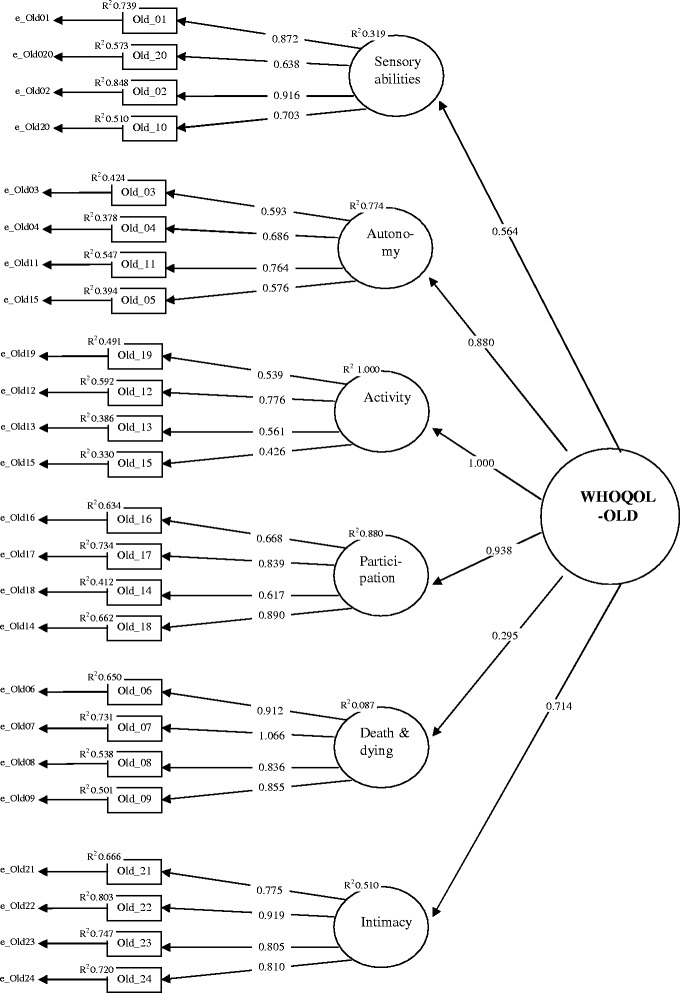
**Confirmatory second order factor model for the six WHOQOL-OLD facets.**

To test this assumption, a second order confirmatory factor model was estimated. For this purpose, the variance of the factor representing the WHOQOL-OLD facet *past, present and future activities* was fixed to zero. The factor loading structure of this model (Figure 9) reveals sufficient standardized loadings above 0.5 of the common factor on five of the six factors representing the WHOQOL-OLD facets. Only the loading on the factor representing the WHOQOL-OLD facet *death and dying* is 0.295, which is far below the limit of 0.500. Moreover, the R^2^ of 0.087 indicates an insufficient low communality for the factor representing the facet *death and dying* but with an estimate of 0.257. This also holds for the factor representing the WHOQOL-OLD facet *sensory abilities*.

The fit-characteristics for both models are presented in Table 5. The Chi^2^ values indicate significant deviances from the empirical covariance structure but that would be expected because of the large sample size. The general fit parameters CFI and TFI are sufficient for both models; the same is true for RMSEA and the SRMR. The comparison of the fit parameters between both models reveals no improvement of the model fit by adding the second order common factor. The loadings clearly show that a one-dimensional representation cannot be recommended (Figure 9).

**Table 5:** **Model fit characteristics of the first order and the second order confirmatory factor models for the WHOQOL-OLD facets**

**Parameter**	**First order factor model**	**Second order factor model**
Chi2	897.86	941.62
Degrees of freedom	237	247
P	0.000	0.000
CFI	0.946	0.943
TLI	0.937	0.937
RMSEA (90% CI)	0.050 (0.046 0.053)	0.050 (0.046 0.053)
Prob. RMSEA < = 0,05	0.565	0.528
SRMR	0.044	0.047
Akaike Information criterion (AIC)	60733.52	60767.70
Bayes Information criterion(BIC)	61171.38	61155.22
Adjusted BIC	60895.02	60910.65

#### Convergent validity

Table 6 shows the correlations between the WHOQOL-OLD facets and the criterion variables. With the exception of the *death and dying* facet, all WHOQOL-OLD facets and the WHOQOL-OLD total score show medium to high positive correlations (between r = 0.363 and r = 0.798) with the WHOQOL-BREF subscales and the WHOQOL-BREF overall score. Medium to high positive correlations were also found between the WHOQOL-OLD facets except *death and dying* and the SF12 subscales “Physical Health Index” and “Mental Health Index.” In contrast to all other WHOQOL-OLD facets, the facet *death and dying* shows much smaller correlations between r = 0.185 and r = 0.286 with the generic quality of life scales.

**Table 6:** **Person correlations of WHOQOL-OLD facets with criterion variables**

	**WHOQOL-OLD facets**						
**Criterion variable**	**Sensory abilities**	**Autonomy**	**Past, present and future activities**	**Social participation**	**Death and dying**	**Intimacy**	**OLD**
**WHOQOL-BREF Physical**	0.589***	0.622***	0.631***	0.737***	0.269***	0.434***	0.738***
**WHOQOL-BREF Psychological**	0.553***	0.666***	0.729***	0.747***	0.286***	0.578***	0.798***
**WHOQOL-BREF Social**	0.363***	0.518***	0.616***	0.590***	0.240***	0.657***	0.674***
**WHOQOL-BREF Environment**	0.459***	0.656***	0.660***	0.699***	0.185***	0.576***	0.722***
**WHOQOL-BREF Overall**	0.486***	0.518***	0.595***	0.647***	0.231***	0.414***	0.648***
**SF12 PHS**	0.536***	0.494***	0.484***	0.593***	0.213***	0.313***	0.592***
**SF12 MHS**	0.395***	0.514***	0.553***	0.538***	0.281***	0.478***	0.623***

*p ≤ 0.05, **p ≤ 0.01, ***p ≤ 0.001.

#### Discriminant validity

Results of the linear regression models are presented in Table 7. As indicated by the standardized regression coefficients, depressive symptoms have the strongest negative effect on all six WHOQOL-OLD facets and on the total WHOQOL-OLD score. The level of cognitive functioning has a positive effect on all facets except *death and dying* and on the total score. The number of chronic diseases is negatively related to *sensory abilities* and to *death and dying* and positively related to *intimacy*.

**Table 7:** **Linear regression models for the WHOQOL-OLD facets (standardized beta coefficients)**

	**Sensory abilities**	**Autonomy**	**Past, present and future activities**	**Social participation**	**Death and dying**	**Intimacy**	**OLD**
**Age**	-.275***	-.011	.108***	.008	.033	.034	-.027
**Female sex**	.048	-.072*	-.013	-.038	-.036	.027	-.019
**Living with others**	-.064	-.060	-.003	.017	.131*	.282***	.081*
**Higher education**	-.005	-.012	.075*	.020	-.048	-.005	.000
**Married**	-.046	.037	-.024	-.012	.098	-.039	.007
**GDS**	-.406***	-.581***	-.707***	-.665***	-.289***	-.576***	-.722***
**IADL**	.035	.088*	-.046	.114***	-.141***	-.013	.003
**No. of chronic Diseases**	-.121***	-.000	.016	-.017	-.184***	.109***	-.053*
**DemTect**	.099***	.141***	.113***	.116***	-.015	.054*	.110***
**N**	956	956	956	956	956	956	956
**Adjusted R** ^**2**^	0.44	0.44	0.52	0.59	0.15	0.45	0.66

*p ≤ 0.05, **p ≤ 0.01, ***p ≤ 0.001.

Socio-demographic characteristics and living arrangements affect only some of the WHOQOL-OLD facets. Increasing age is related to decreasing *sensory abilities* but positively to *past, present and future activities*. Female sex is negatively related to *autonomy*. In comparison to persons who live alone, those who live with others have a higher quality of life on the WHOQOL-OLD facets *death and dying*, *intimacy* and a higher WHOQOL-OLD total score. Persons with a higher formal education assess their *past, present and future activities* better than those with a lower educational level.

As indicated by the adjusted R^2^ a considerable amount of variance was explained by the model variables.

## Discussion

This is the first examination of the psychometric properties of the WHOQOL-OLD for a representative sample of the German population aged 60 years and older. Psychometric properties were examined by means of the classic test theory and, essentially, by probabilistic test theory.

The examination of the parameters for the internal consistency revealed high reliability coefficients and high item-scale respective intern item correlations for four facets *sensory abilities*, *participation*, *death and dying* and *intimacy* of the six facets of the WHOQOL-OLD. The remaining two facets *autonomy* and *activity* show low, but still acceptable, values for the internal consistency.

Results of the probabilistic test theory approach indicate that all facets of the WHOQOL-OLD can be represented by a partial credit model with ordered thresholds. Fit indices show that all items are productive for measurement. The thresholds of the answering categories have an ascending order for all items but the varying thresholds between the answering categories indicate that the measurement characteristics of the items and the answering categories are unequal.

The construct validity of the six-facet model of the WHOQOL-OLD was supported by the first order confirmatory factor analysis for the six facets model but not by the second order model for the WHOQOL-OLD total scale.

Convergent validity of the WHOQOL-OLD facets could be well confirmed with regard to the subscales of the generic quality of life measures WHOQOL-BREF and SF12.

Results from the multiple regression models indicate that symptoms of depression are the strongest predictor of all WHOQOL-OLD facets. Nevertheless, cognitive functioning, the ability to carry out daily activities and chronic diseases are also important factors in explaining quality of life.

Results of our analyses reveal that the psychometric properties of the German version of the WHOQOL-OLD are similar, as good as, or better than those reported from the international WHOQOL-OLD field study [51] and as those of other country versions recently tested in Norway [52], China [21], Brazil [53,54], France [55] and Turkey [56].

As revealed by Power et al. [51] for the international WHOQOL-OLD data set and by Liu et al [57] for the Chinese version of the WHOQOL-OLD, a good construct validity was obtained for the German version of the WHOQOL-OLD in our study for the six facet structure but not for second order factor model. These results underline that the WHOQOL-OLD represents a multidimensional construct of quality of life in old age that cannot be reduced to one latent dimension. Nevertheless, efforts have been made to develop a short version of the WHOQOL-OLD [57] and the authors recommend three versions with different selections of six items from the WHOQOL-OLD. However, the reliability of all three versions of this instrument is worse in comparison to that of the WHOQOL-OLD.

As in the cross-cultural WHOQOL-OLD studies [51] and in several national studies [21,58-60], depressive symptoms were also found to explain a considerable amount of variance in all facets of the German version. Chachamovic et al. [60] examined the effects of a major depression diagnosis in comparison to subclinical symptoms of depression and found that even in the absence of a diagnosis of a major depression, sub clinical symptoms of depression have a strong negative effect on all facets of the WHOQOL-OLD.

The strong negative effect of depressive symptoms on QoL in the German population corresponds with results from cross cultural studies on the importance of different domains of QoL showing that the presence of positive feelings and the absence of negative feelings ranked higher than average in the German sample [61]. The importance of positive feelings on QoL could be related to the high level of economic development in Germany. Economic development has been identified as a major cultural factor in explaining the variance in cross cultural importance rankings. While in developing countries the facets related to physical health were ranked higher than those related to psychological well-being, the opposite was the case in developed countries [61]. Nevertheless, associations of important rankings of psychological well-being with economical development do not necessarily result in different effects of depressive symptoms on QoL. Dragomirecka et al. [62] identified depressive symptoms as the main predictors of most WHOQOL-OLD domains in all countries independent of the countries’ economical wealth status in their cross cultural comparison of QoL in the elderly population of six European countries [62]. These results support the hypothesis that depressive symptoms are intercultural predictors of quality of life in elderly people. However, since most studies on QoL in elderly people are cross-sectional, the exact relationships between objective living circumstances, cultural factors, depressive symptoms and QoL are still unclear. Longitudinal cross-cultural studies would allow for the analysis of whether cultural factors or symptoms of depression work as mediator or moderator variables in this relationship.

### Limitations

Due to the cross-sectional design of the study, test-retest reliability and sensitivity to change of the WHOQOL-OLD could not be assessed. The clinical status of the respondents was assessed by means of the self-rating GDS, which does not allow the diagnosis of major depression. Therefore, it was not possible to examine differences between the impact of clinical and sub-clinical levels of depression.

## Abbreviations

WHOQOL: World Health Organization Quality of Life
GDS: Geriatric depression scale
IADL: Instrumental activities of daily living
IRT: Item response theory
MCI: Mild cognitive impairment
PCM: Partial credit model
PSI: Person-separation-index
PIM: Person-item map
SRMR: Standardized root mean square residual
RMSEA: Root mean square error of approximation
CFI: Comparative fit index
TLI: Tucker-lewis index
